# Effects of siRNA silencing on the susceptibility of the fish cell line CHSE-214 to *Yersinia ruckeri*

**DOI:** 10.1186/s13567-020-00760-6

**Published:** 2020-03-20

**Authors:** Simon Menanteau-Ledouble, Oskar Schachner, Mark L. Lawrence, Mansour El-Matbouli

**Affiliations:** 1 Clinical Division of Fish Medicine, University of Veterinary Medicine, Vienna, Austria; 2 Feed the Future Fish Innovation Lab for Fish, Mississippi State, MS USA

## Abstract

*Yersinia ruckeri* is a facultative intracellular enterobacterium mostly known as the causative agent of enteric redmouth disease in salmonid fish. In the present study, we applied RNA inhibition to silence twenty pre-selected genes on the genome of a fish cell line (CHSE-214) followed by a gentamicin assay to quantify the effect of silencing on the cells’ susceptibility to infection and found that silencing of 18 out of 20 genes significantly reduced the number of *Y. ruckeri* recovered. These findings improve our understanding of the infection process by *Y. ruckeri* and of the interactions between this bacterial pathogen and host cells.

## Introduction, methods and results

The enterobacterium *Yersinia ruckeri* is a major fish pathogen worldwide and has mostly been studied as the causative agent of enteric red-mouth disease in salmonid fish [1, 2]. Several virulence factors have been described in *Y. ruckeri* (see reviews by Kumar et al. and Wrobel et al. [1, 3]). *Y. ruckeri* causes septicaemia and haemorrhages leading to high levels of mortality in infected fish [1, 2]. The bacterium also has zoonotic potential and has been associated with topical infections in humans [4]. Like several other members of the genus *Yersinia*, *Y. ruckeri* has demonstrated the ability to invade non-professional phagocytic cells [5, 6], allowing the bacterium to access restricted nutrients and protecting it from the immune system. It might help the bacterium to cross epithelial membranes as is the case for other *Yersiniaceae*.

Two main mechanisms of entry have been described in bacteria, including *Yersiniaceae*. The zipper mechanism is initiated by the binding of the bacterial adhesins to specific molecules on the cell membrane (see the review on yersinia adhesins by Chauhan et al. [2]). In *Y. pseudotuberculosis* and *Y. enterocolitica*, two autotransporter proteins invasin (Inv) and Yersinia adhesin A (YadA) interact with integrin receptors [7, 8]. Interactions of these bacterial proteins with receptors on the surface of the host’s cells lead to the recruitment of more receptors, activation of Rac1 and cytoskeletal rearrangement culminating in the uptake of the bacterium [9]. Interestingly; while *Y. ruckeri* does not harbour a YadA equivalent, it is known to harbour two homologs of invasin: *Y. ruckeri* invasin (YrInv) and *Y. ruckeri* invasin-like molecule (YrIlm) [3]. Furthermore, several isolates of *Y. ruckeri* belonging to the O1 serotype also carry a cluster of fimbrial gene homologous to the STF cluster of *S. typhimurium* [10, 11]. However, this cluster is absent from the ATCC 29473 type strain studied here [11].

The other main mechanism of entry studied is the trigger mechanism that relies on effector proteins secreted through the type three and type four secretion system (T3SS and T4SS). Once inside the host’s cells, these effector proteins interact with regulatory proteins in the host, in particular members of the Rho family (RhoGTPases Rac, Cdc42 or RhoG) [12], leading to a rearrangement of the cytoskeleton of the host cells and the uptake of the bacterium [12]. Intriguingly, the T3SS of *Y. ruckeri* actually belongs to the Ysa family, a different family from the one most studied in *Yersiniaceae* and is more closely related to the T3SS carried on *Salmonella* pathogenicity island 1 (SPI-1) of *Salmonella enterica* [13, 14]. Among the proteins carried on SPI-1 is the chaperon Invasion protein B (InvB); SPI-1 is known to play a role in the intracellular invasion of *S. enterica* [15] so the Ysa of *Y. ruckeri* could plausibly be involved in intracellular invasion of *Y. ruckeri.* However, our knowledge of *Ysa* T3SS, and that of *Y. ruckeri* in particular is still very incomplete [10] and no conclusion is currently possible.

An important feature of both invasion mechanisms is that they require active uptake of the bacterium by the cell and it is possible to prevent host cells from internalising the bacteria, for example by treating them with chemical blockers [5, 6]. Similarly, silencing of host genes has been shown to inhibit bacterial internalisation. For example, 305 host genes have been associated with the ability of *Listeria monocytogenes* to invade cell cultures of drosophila SL2 cells and 86 genes necessary for invasion by *Mycobacterium fortuitum* [16]. In human macrophages, 270 molecules have been identified whose silencing affected the bacterial load of seven different field isolates of *Mycobacterium tuberculosis* [17]. Finally, 252 genes have been shown to be involved in the survival of *Salmonella enterica* serovar *Typhimurium* within the human epithelial cell line MCF-7 [18]. Among these genes, the most impactful were SEC22A, Rab1B and VPS33B that were involved in vesicle trafficking as well as ATP6VOD1, an ATPase involved in vacuole acidification and the iron transporter FTHL17. Interestingly, the authors noted a significant overlap between their findings and that reported by Kumar et al. for *M. tuberculosis* [17] suggesting that many of these targets are well conserved even between very evolutionary distant bacteria.

In the present study, we aimed to produce siRNA to silence 20 genes commonly involved in invasion by bacterial pathogens (see Additional file 1), including surface integrins since such molecules are often targeted by the invasin molecules expressed by other *Yersiniaceae* [2]. Other genes were cytoskeletal molecules and genes involved in vacuolar trafficking and maturation [17, 18] as well as agents of the cytoskeletal apparatus since this plays a central part in the internalization of bacteria through both zipper and trigger mechanisms [12]. The selection also included genes and pathways shared by *L. monocytogenes*, *M. tuberculosis* as well as *S. typhimurium* [16–18] since these pathways are used by such diverse facultative intracellular pathogens, they might also be involved in the intracellular invasion of *Y. ruckeri*.

The fish cell line Chinook Salmon Embryo (CHSE-214) was used since we wanted to investigate an epithelial cell line derived from salmonids. In previous experiments using multiple cell lines we showed that it was well suited for invasion by *Y. ruckeri*. PCR and sequencing were applied to confirm that the CHSE214 cells were indeed derived from Chinook salmon (*Oncorhynchus tshawytscha*). Similarly; the study focussed on the type strain ATCC 29473, a type strain belonging to biotype 1, since these experiments showed that it had a high potential for invasiveness [5].

### siRNA transfection

Sequences for the siRNA were designed using the Silencer Select siRNA design algorithm. For the genes for which no *O. tshawytscha* sequences were available, sequences from other members of the *Oncorhynchus* genomes were used. Upon reception, the siRNA were resuspended to 20 µM and stored at −20 °C until use, according to the manufacturer’s instructions.

CHSE-214 cells were grown in 24 well plates at 20 °C supplied with 1250 µL Minimum Essential Medium (MEM-glutaMAX™, Gibco, Thermo-Fisher Scientific, Waltham, USA) containing 2% FBS, where they reached about 80% confluence 1 day after seeding. On the day of the assay, 8 µL of the siRNA solution was added to 400 µL of Opti-MEM medium (Gibco). Seventy-two microliters of RNAiMax lipofectamine reagent (Sigma Aldrich, St Louis, USA) was diluted in 1200 µL of Opti-MEM medium. The two solutions were mixed and incubated 5 min at room temperature. Afterwards, the culture medium on top of two adjacent rows of the 24 wells was replaced with 50 µL of the solution and, after 20 min, 450 µL of fresh MEM-Glutamax medium was added.

Ambion^®^*Silencer*^*®*^ Negative Control #1 (Thermo-Fischer Scientific) was applied as a negative control. The cells were incubated at 20 °C and since the temperature commonly used for transfection was lower than for other cell lines, incubation time was extended to 3 days.

### Bacterial invasion assay

The gentamicin assay was performed as previously described [5]. Briefly, *Y. ruckeri* ATCC 29473 was cultivated overnight in brain heart infusion (BHI, Oxoid, Thermo-Fisher Scientific). Optical density of the culture was assessed by spectrophotometry (Eppendorf Biophotometer, Hamburg, Germany) and adjusted to an optical density of .5 at 600 nm. Bacteria were pelleted by centrifugation at 3220 *g* and 20 °C (in an Eppendorf Centrifuge 5810 R) and resuspended in 10 times the original volume of MEM-Glutamax. Then, the culture medium in the leftmost row was replaced by this bacterial solution and the bacteria were left to interact with the cells for 5 h at room temperature. In the other row, the medium was replaced by fresh MEM-glutamax without bacteria. After that time, the medium was removed, the cells were washed three times with phosphate buffered saline (PBS) and a fresh volume of MEM-glutamax, supplemented with the antibiotic gentamicin (Sigma-Aldrich) at a concentration of 100 μg/mL was added to the wells. The antibiotic was left to act for 4 h. Afterwards the cells were washed twice with PBS before replacing the medium supplemented with 1% Triton-X (Sigma-Aldrich). After 10 min of exposure to the detergent, the cells were triturated with a micropipette and serially diluted from 10^−1^ to 10^−4^ before being plated onto Brain heart infusion agar (BHIA, Oxoid). Each of the three wells represented a biological replicate and each dilution was plated in technical quadruplicates, meaning that 48 plates were inoculated for each siRNA. The agar plates were incubated at 22 °C until clear colonies were visible and counted (generally after 48 h) and the average CFU per mL value was calculated.

### Controls

For each siRNA, the cells numbered 1D, 3D and 5D were left un-inoculated in each 24 well plate then lysed and plated to act as a negative control and detect any contamination of the reagents. Similarly, the cells in the wells 2D, 4D and 6D were also left un-exposed to the bacteria and a trypan blue assay was performed to rule-out a toxic effect of the siRNA silencing: The cells were washed three times in PBS, then .2% Trypan Blue (Gibco, .4% diluted 1:1 in PBS) was added. After 1 min, fixation was performed with 4% formalin for 10 min. The cells were then rinsed until any trace of blue dye had disappeared. The plates were kept at 4 °C until quantification of the cells using an inverted microscope (Leica DM IRB, Wetzlar, Germany). One hundred cells were counted and the number of blue stained cells among them was recorded. The procedure was repeated 4 more times for each culture unit to result in 5 percentage values for each siRNA. These were then compared to the survival of the control cells without siRNA to confirm that the silencing procedure did not have a toxic effect. In no instances did these numbers differ significantly from that of the control.

### Preparation of the cDNA samples

Finally, the cells in the rightmost row for each treatment were lysed in buffer RLT (Qiagen, Hilden, Germany). The cell suspension was homogenised using QIAshredder columns (Qiagen) and centrifugation at 145 000 RPM for two minutes at room temperature using MiniSpin tabletop centrifuge (Eppendorf). Afterwards, the RNA were extracted using the Rneasy mini kit (Qiagen). RNA were immediately quantified using a Nanodrop machine (Thermo-Fisher) and cDNA were immediately synthesised using Iscript kits (Bio-Rad) in a C1000 Touch thermocycler (Bio-Rad, Hercules, USA). cDNA were stored at 4 °C until use.

### RT-qPCR

After obtention of these cDNA, RT-qPCR were performed to compare the level of expression between the genes targeted by siRNA and the control and confirm that the siRNA had successfully silenced the expression of these genes. The primer sequences used are listed in Table 1 and ubiquitin and elongation factor 1-alpha primers designed by Peña et al. [19] were used for the housekeeping reference genes.

**Table 1:** **Primer sequences for the RT-qPCR confirmation of the siRNA silencing**

Ubiquitin*	GGAAAACCATCACCCTTGAG*
	ATAATGCCTCCACGAAGACG*
Elongation Factor 1 alpha*	GTCTACAAAATCGGCGGTAT*
	CTTGACGGACACGTTCTTGA*
Protein kinase C	TTGTTTGGCGGACTCCTGAA
	CCACGCCGAAAACAATCTCC
Rab-1A	AACCCCACCAACACCTCTAC
	GCACACACACACAACTTCTTTC
SEC22b-B	AGAGAGGAGAGGAGATAGGG
	TGGATAGAATGTGAGACCAGA
Vacuolar ATP synthase subunit A	CCGCTGAGGACTTCCGATAAAC
	ACAGCCTTCCATAGCATTGCAC
VPS-associated protein	ATCCACAACCTGACTGCCTACC
	CTCTCGCTGCTCTTGATGAAC
Rho1-GTPase	TAGCCCCAGTTTGCTCTTCG
	GCTGTCTCGTTCACTGGTCA
Ube213	AACCCCACCTCCTTTCTCTC
	TTCACTAAGTCCCCTTGTTCC
Sumo 2	TCCACCACAGAACCAATACC
	CAAAACAGGAAGGAAACAAAGG
Equilibrative nucleoside transporter 1	ACGTCCGTGTTAAATGCTC
	ACAAAATCTCTCCTCCCCTC
Integrin β-1 precursor	ACCAATCCTACCCCCTATCC
	AGACCTCCCTCATTGTGTCG
Actin	CCAAGCAGGAATACGACGAG
	GCGTGGCAAAAAAAGTCCAG
Rac1	ACTCACCCCCATTACCTACC
	CTGCTCCAATCTCTTTTGCC
SDC42	AGAGGGGAAGGAATCAACAG
	ATGACAGGCAGGTAGGAGAG
Rho GTPase-activating prot.	CAGAAGAAAATGAGCAAAGACC
	CCTGAGAACTACTGTCCACC
Laminin 2	GCGAATCAGAACCCTCAAC
	GCAGATACACATCCCTCCAAC
β-Cadherin	TCCCTCAGTTCCCTCAACTC
	TCTCAATCCTCTCCTCCTCC
Myotubularin-related protein 2	TCAAGCGGAATACAAAAGACAG
	CCACTCTCTTCAACTCCTCATC
MaPK	TTCACTTCTCAAACCCCAAAC
	CGCTCAAAGGACAAACCAC
Caspase I precursor	GTGGAACAACAGGAAACAAAG
	TGATGGCAGTAGAACAGGG
Cyclin D1	GCTGTCCTGTATGCTCTCTC
	CCCAAACCATTCCATTCTTCTC

The two first (with an asterisk) primer pairs are the housekeeping genes and were designed by Peña et al. [19].

Genomic DNA was extracted from CHSE-214 cells using the DNeasy kit (Qiagen) according to the manufacturer’s instructions. End point PCR was performed using these primers to confirm the optimal annealing temperature, afterwards the PCR products were purified with a QIAquick gel purification kit (Qiagen). Products’ concentrations were measured using a nanodrop, then adjusted to 10 ng/µL. Afterwards, serial dilution was performed to produce concentrations ranging from 10^−1^ to 10^−4^ ng/µL. qPCR were then performed on these serial dilutions as well as the cDNA produced as described in the previous section. Relative gene expression levels of the silenced genes were calculated using the 2^−ΔΔCt^ method and converted into percentage for clarity in order to confirm the efficacy of the silencing. Serial dilutions were used to calculate the R^2^ and efficiency of the qPCR and confirm that these were above .9 and between 95 and 100%, respectively for every qPCR.

## Results

RT-qPCR confirmed the efficiency of the silencing since all treated genes had a lower expression level compared to the control (see Additional file 2).

Silencing of every one of the twenty tested genes resulted in some reduction of the number of bacteria recovered from CHSE-214 cells at the end of the assay. However, this reduction was only statistically significant in 18 out of 20 genes (Table 2).

**Table 2:** **Genes investigated in this study and silencing effect**

Gene	Average number of bacteria recovered	Mean Difference in number of bacteria recovered compared with control	Sig. of the effect of the silencing	Number of bacteria recovered as percentage of those recovered in the untreated control (%)
Scrambled RNA control	3.13E+05			100.0
*Sec22B*	1.05E+05	2.08E+05*	.004	33.4
*ATPAse subunit F*	1.05E+05	2.07E+05*	.001	33.7
*VPS11*	5.96E+04	2.53E+05*	.001	19.1
*Rho GTPase*	1.27E+05	1.86E+05*	.012	40.5
*Ube2EL3*	1.74E+05	1.39E+05^a^	.831^a^	55.7
*sumo2*	2.98E+04	2.83E+05*	.001	9.5
*Nucleoside transporter 1*	1.54E+04	2.97E+05*	.001	4.9
*Integrin beta 1*	1.57E+04	2.97E+05*	.001	5.0
*Actin*	1.98E+04	2.93E+05*	.001	6.3
*Rac1*	9.83E+03	3.03E+05*	.001	3.1
*CDC42*	2.78E+04	2.85E+05*	.001	8.9
*arhgap18*	2.61E+04	2.87E+05*	.001	8.3
*lama2*	9.43E+03	3.03E+05*	.001	3.0
*β*-*Cadherin*	3.04E+04	2.82E+05*	.001	9.7
*Myotubularin protein 2*	1.17E+04	3.01E+05*	.001	3.8
*MApk14A*	2.70E+04	2.86E+05*	.001	8.6
*Caspase I precursor*	1.85E+05	1.28E+05^a^	1.000^a^	59.1
*Cyclin D1*	1.03E+05	2.09E+05*	.005	33.1
*ProtKinC*	2.91E+04	2.84E+05*	.001	9.3
*Rab1*	3.54E+04	2.77E+05*	.001	11.3
Total	8.22E+04			

Silencing effect was determined by comparing the number of bacteria recovered at the end of the gentamicin assay. Significant difference between the results and the control are further indicated with an asterisk.

^a^Genes (ubiquitin conjugating enzyme Ube2L3 and caspase I precursor) represent genes whose silencing did not significantly impact the gentamicin assay.

The two genes whose silencing had the strongest statistical effect were *lama2* which encodes a homolog of the extracellular matrix protein laminin subunit α-2 (Laminin 2) that play an important role in cell adhesion as well as the *Ras*-*related C3 botulinum toxin substrate 1* (Rac1) gene that encodes an important regulatory G protein. Silencing of these genes reduced the number of surviving bacteria to 3.0 and 3.1% of the corresponding control, respectively (*P* < 0.001).

Other genes whose silencing had a great impact included the myotubularin protein 2 and the Equilibrative nucleoside transporter 1, a glycoprotein that mediates the cellular uptake of nucleosides from the environment (3.8% and 4.9% compared to the control, respectively; *P* < 0.001). Followed by the precursor for the transmembrane receptor integrin β-1 that acts alongside integrin alpha 1 and integrin alpha 2 to form collagen receptors involved in cell adhesion (5.0% of control, *P* < 0.001) and actin (6.3% compared to control; *P* < 0.001). Finally, three more notable genes included the one encoding for the regulator protein kinase C (9.3% of the control; *P* < 0.001), alongside *sumo2*, that encodes a ubiquitin like-protein that binds to target protein as part of a post-translational modification system and plays a role in nuclear transport (9.3% of the control; *P* < 0.001) as well as the gene encoding the Ras related protein Rab1A that is involved in the regulation of vesicular transport, in particular in relation with the Golgi apparatus (11.3% compared to the control; *P* < 0.001).

Conversely, the two genes whose silencing had no significant effect on the bacterial invasion included the gene encoding the caspase I precursor; caspase I plays an important role in cellular immunity by activating immune-related proteins such as interleukin by proteolytic cleavage (59.1% of the control, *P* = 1.000). The second gene encoded the ubiquitin conjugating enzyme Ube2L3 (55.7% of the control; *P* = .831) that contributes to the ubiquitination of target proteins prior to degradation.

## Discussion

### Genes whose silencing had the most significant effect

*Lama2* was the gene whose silencing had the strongest statistical effect. This result is comparable to that of van Wijk et al. who reported that silencing of *lama2* protected hamster ovary (CHO) cells from invasion by several isolates of *Streptococcus* and *Staphylococcus aureus* [20]. Previously, a modified Western-blot procedure had also shown that Lama2 was a substrate for the adhesion of *Y. enterocolitica*, including for isolates of the bacterium that did not express YadA, demonstrating that this binding was YadA independent [21]. This is particularly relevant as YadA is also absent from *Y. ruckeri* and it is therefore plausible that laminin could constitute an important substrate for the binding of *Y. ruckeri*’s adhesins.

Silencing of the Rac1 gene had an equally strong effect. Rac1 is a member of the Rho family of GTPases and plays a central role regulating cytoskeleton rearrangement. This regulator has previously been involved in intracellular invasion of the enterobacteriaceae *Salmonella typhimurium* [22] and is targeted by the effector protein IpgB1 of *Shigella* [23]. In a previous experiment, we reported that the inhibitor of Rac1 N-acetylcysteine completely inhibited entrance of *Y. ruckeri* into CHSE-214 cells [5], which is very consistent with our current findings.

Another gene whose silencing had a high effect was the myotubularin protein 2. Myotubularins are tyrosine phosphatases and, while their role during bacterial infections has been poorly characterised, genome wide screening of Thornbrough et al. showed that silencing myotubularin protein 3 protected Michigan Cancer Foundation-7 (MCF-7) cells from infection by *Salmonella typhimurium* [18]. Indeed, among the 252 susceptibility factors identified in this study, myotubularin protein 3 was among the ones that had the strongest impact on intracellular bacterial infection [18].

Similarly, Equilibrative nucleoside transporter 1 is a member of a well-conserved family of transmembrane proteins. Pathogenic intracellular bacteria are known to scavenge nucleotide from their environment and therefore a reduction in the nutrients available to the bacterium could explain the effects of silencing this gene.

Another molecule whose silencing had a significant effect on the cells’ sensitivity to infection was the precursor for integrin β-1. Integrins are trans-membrane receptors and their role in cell adhesion to the extra-cellular matrix is well known. Moreover, integrins β-1 have been well studied as targets of the adhesin molecules of *Yersinia* sp., including *Y. pseudotuberculosis* [24] and *Y. enterocolitica* [25]. As such, they play a central role in the use of the zipper mechanisms by these bacteria to gain intracellular entry. Notably, *Y. ruckeri* possesses two adhesins homologous to invasin (YrInv and Yrilm) [3]. The finding that this target is also important for *Y. ruckeri* suggests that this bacterium might gain intracellular entry through the zipper mechanism, targeting integrin-β molecules using invasin-like adhesins, in a manner similar to that of *Y. pseudotuberculosis* and *Y. enterocolitica*.

Similarly, silencing of the actin gene also had a very notable effect. Actin is a central component of the cytoskeleton that plays a critical role in both the trigger and zipper mechanism [12].

Finally, two more genes whose silencing had a statistically significant effect were the genes coding for the protein kinase C, a regulatory protein overseeing a large number of varied functions, including signal transduction as well as the expression of the cytoplasmic tyrosine kinase, Focal Adhesion Kinase (FAK) and actin rearrangement [26]. Previously, protein kinase C activity has shown an inverse correlation with the uptake of *L. monocytogenes* by murine macrophages, as well as a positive correlation with the escape of these bacteria from the macrophage endosomes [27] and it has been shown that protein kinase C recruitment in lipid rafts was induced by Enterohemorrhagic *E. coli* O157:H7. In the present study, silencing of the gene encoding for protein kinase C resulted in a strong decrease of the cell susceptibility to bacterial infection (*P* < 0.001).

Finally, silencing of the Rab1A molecule also had a notable effect on the cell’s susceptibility to *Y. ruckeri* infection. Rab1A is a member of the Rab family, small GTPases acting together as network involved in the regulation of vesicular transport. Rab1A is specifically required for the microtubule-based motility of murine endocytic vesicles [28] and silencing of Rab1b was shown to lead to a reduced growth of *S. enterica Typhimurium* within MCF-7 cells [18]. Rab1A’s isoform Rab1b is also known to be necessary for the survival of *Y. pestis* within murine macrophages [29].

### Genes whose silencing had no significant effect

Two genes were found for which silencing did not result in a significant reduction in the number of recovered bacteria. The first was the caspase I precursor. Caspases take part in the cellular immune response and play an important role in regulating apoptosis and Caspase I is known to be inhibited by the YopJ effector molecule of *Y. pestis* [30]. If it is similarly targeted by a virulence factor of *Y. ruckeri*, this could explain why it is not necessary for this bacterium’s invasion.

Finally, the other gene whose silencing had no statistically significant effect was the gene coding for the ubiquitin conjugating enzyme Ube2L3 (55.7% of the control; *P* = .831). A similar protein, Ube2L6 was among the new factors reported by Thornbrough et al. as involved in the intracellular infection by *S. typhimurium* [18]. Ube2L3 is also known to be a substrate for the effector protein YopM of *Y. pseudotuberculosis*, however, there was no other information to suggest a similar involvement of Ube2L3 in the invasion of *Y. ruckeri*.

## Supplementary information

**Additional file 1. Genes selected for this experiment with a brief description of their expected role in bacterial invasion and the rational for their selection.**

**Additional file 2. Relative gene expression of the silenced genes compared to the control, as assessed by RT-qPCR.** Relative gene expression levels of the silenced genes were calculated using the 2^−ΔΔCt^ method and ubiquitin and elongation factor 1-alpha were used as reference genes according to Peña et al. [19]. Average results compared to both house-keeping genes were then calculated and relative expression values were then converted into percentage of the expression into the silenced genes compared to the control for clarity. Greyed out cells represent genes whose silencing did not significantly impact the gentamycin assay.

## Abbreviations

Ail: Attachment invasion locus
BHI: Brain heart infusion
cDNA: Complementary DNA
CDC42: Cell division cycle 42
CHSE-214: Chinook Salmon Embryo
CFU: Colony forming unit
FAK: Focal adhesion kinase
FTHL17: Ferritin heavy chain like 17
MCF7: Michigan Cancer Foundation-7 cell line
MEM: Minimum essential medium
MS222: Tricaine methanesulfonate
Rac1: Ras-related C3 botulinum toxin substrate 1
RPM: Rotation per minute
RT-qPCR: Reverse transcription quantitative real-time PCR
SEC22A: Secretory protein 22A
siRNA: Small interfering ribonucleic acid
SPI1: Salmonella pathogenicity island 1
T3SS: Type 3 Secretion System
T4SS: Type 4 Secretion System
VPS33B: Vacuolar protein sorting-associated protein 33B
Yop: *Yersinia* outer protein
YrInv: *Yersinia ruckeri* invasion
YrIlm: *Yersinia ruckeri* invasin-like molecule
