# Supplementary material for: Effects of siRNA silencing on the susceptibility of the fish cell line CHSE-214 to Yersinia ruckeri
Source: Vet Res. 2020 Mar 20;51:45. doi: 10.1186/s13567-020-00760-6 (PMC7083013; doi:10.1186/s13567-020-00760-6)
Supplement: Supplementary file 1 — Additional file 1. Genes selected for this experiment with a brief description of their expected role in bacterial invasion and the rational for their selection. [file 13567_2020_760_MOESM1_ESM.docx]

**Additional file 1 Genes selected for this experiment with a brief description of their expected role in bacterial invasion and the rational for their selection.**

| **Gene** | Role and rationale for inclusion | Ref.: |
| --- | --- | --- |
| ***protein Kinase C*** | Activator of Protein Kinase C among the molecules required for intracellular survival of *Salmonella* | [18] |
| ***Rab-1A*** | Involvement in intracellular growth of *Salmonella* and other bacterial pathogens | [16, 18] |
| ***Sec22B*** | Involvement in intracellular growth of *Salmonella* | [18] |
| ***vacuolar ATPAse Subunit F*** | Role in vacuolar acidification that is necessary for intracellular survival of *Salmonella* | [18] |
| ***VPS-associated protein 11*** | Vaculoar sorting, involved in intracellular growth of *Salmonella* and *M. tuberculosis* | [17, 18] |
| ***Rho GTPase*** | Mitochondrial RhoGTPase necessary for intracellular survival of *Salmonella* | [16, 18] |
| ***ubiquitin conjugating enzyme Ube213*** | Ubiquitin, involvement in intracellular growth of *Salmonella* | [18] |
| ***Sumo2*** | Post-translational modification system and nuclear transport | [16] |
| ***equilibrative nucleoside transporter 1*** | Homolog of SLC29A required for intracellular growth of *Salmonella* | [18] |
| ***integrin Beta 1*** | Common substrate for Yersinial adhesins | [2] |
| ***actin*** | Central molecule of the cytoskeleton | [9] |
| ***Rac1*** | Chemical inhibition of Rac1 prevented infection by *Y. ruckeri* | [5] |
| ***CDC42*** | Role in activation of the JNK pathway | [18] |
| **Rho GTPase-activating protein *18*** | GTPase-activating signal transduction molecule | [16] |
| ***laminin 2*** | Adhesion to basement, necessary for infection by *Streptococcus and Staphilococcus aureus* | [20] |
| ***β-cadherin*** | Receptor for bacterial invasion molecules in particular invasion of *L. monocytogenes* and the adhesins of *Shigella flexneri* | [9] |
| ***myotubularin-related protein 2*** | Involvement in intracellular growth of *Salmonella* | [18] |
| ***MApk14A*** | Part of the MApK cascade | [17] |
| ***caspase I precursor*** | Activated by *Y. pestis* and target of its T3SS. | [30] |
| ***cyclin D1*** | Role in the cell cycle | [17] |
