# Supplementary material for: Effects of siRNA silencing on the susceptibility of the fish cell line CHSE-214 to Yersinia ruckeri
Source: Vet Res. 2020 Mar 20;51:45. doi: 10.1186/s13567-020-00760-6 (PMC7083013; doi:10.1186/s13567-020-00760-6)
Supplement: Supplementary file 2 — Additional file 2. Relative gene expression of the silenced genes compared to the control, as assessed by RT-qPCR. Relative gene expression levels of the silenced genes were calculated using the 2−ΔΔCt method and ubiquitin and elongation factor 1-alpha were used as reference genes according to Peña et al. [19]. Average results compared to both house-keeping genes were then calculated and relative expression values were then converted into percentage of the expression into the silenced genes compared to the control for clarity. Greyed out cells represent genes whose silencing did not significantly impact the gentamycin assay. [file 13567_2020_760_MOESM2_ESM.docx]

**Additional file 2 Relative gene expression of the silenced genes compared to the control, as assessed by RTqPCR.** Relative gene expression levels of the silenced genes were calculated using the 2^-ΔΔCt^ method and ubiquitin and elongation factor 1-alpha were used as reference genes according to Peña et al. [19]. Average results compared to both house-keeping genes were then calculated and relative expression values were then converted into percentage of the expression into the silenced genes compared to the control for clarity. Greyed out cells represent genes whose silencing did not significantly impact the gentamycin assay.

| **Gene name** | **Relative gene expression** |
| --- | --- |
| **Protein kinase C** | 1.1% |
| **Rab-1A** | 26.9% |
| **SEC22b-B** | 20.9% |
| **Vacuolar ATP synthase subunit A** | 11.9% |
| **VPS-associated protein 33A** | 31.8% |
| **Rho1-GTPase** | 15.1% |
| **Ubiquitin conjugating enzyme E2L3** | 9.3% |
| **Sumo 2** | 21.3% |
| **Equilibrative nucleoside transporter 1** | 8.0% |
| **Integrin β-1 precursor** | 9.3% |
| **Actin** | 17.7% |
| **Rac1** | 15.0% |
| **CDC42** | 19.2% |
| **Rho GTPase-activating protein (*arhgap18*)** | 21.7% |
| **Laminin** | 7.2% |
| **β-cadherin** | 14.4% |
| **Myotubularin-related protein 2** | 4.5% |
| ***p38b1 mitogen activated protein kinase*** | 6.2% |
| **Caspase I precursor** | 9.5% |
| **Cyclin D1** | 14.1% |
